# The GenePOC Platform, a Rational Solution for Extreme Point-of-Care Testing

**DOI:** 10.3390/mi7060094

**Published:** 2016-05-24

**Authors:** Luc Bissonnette, Michel G. Bergeron

**Affiliations:** 1 Centre de recherche en infectiologie de l’Université Laval, Axe maladies infectieuses et immunitaires, Centre de recherche du CHU de Québec-Université Laval, Québec City, QC G1V 4G2, Canada; Luc.Bissonnette@crchul.ulaval.ca; 2 Département de microbiologie-infectiologie et d'immunologie, Faculté de médecine, Université Laval, Québec City, QC G1V 0A6, Canada

**Keywords:** extreme point-of-care testing, low-resource settings, emergencies, disasters, infectious disease diagnostics, centripetal (centrifugal) microfluidics, polymerase chain reaction, isothermal amplification, GenePOC

## Abstract

Extreme point-of-care (POC) testing for infections, as performed (endured) in low-resource settings, developing countries, tropical areas, or in conditions following emergency crises or natural disasters, must be undertaken under environmental, logistic, and societal conditions which impose a significant deal of stress on local human populations and healthcare providers. For disease diagnostics or management, simple and robust biomedical equipment and reagents are required and needed. This chapter aims to overview some of these stresses (requirements) and intends to describe some of the solutions already engineered at the heart of centripetal (centrifugal) microfluidic platforms such as that of GenePOC Inc. to enable rapid, robust, and reproducible nucleic acid-based diagnostics of infectious diseases, to better control the morbidity and mortality of infections and the expanding threat posed by antimicrobial resistance.

## 1. Introduction

Nowadays, point-of-care (POC) diagnostics, seldom synonymous of bedside testing, must be envisioned not only as a healthcare technology but rather as a multifocal strategy aiming to better instruct patient management by decreasing both the turn-around time of a test and the distance from the site where the analysis is performed [1,2,3,4,5]. Indeed, POC strategies are rapidly gaining importance in infectious diseases where the critical time window for appropriate management, essentially initiating proper and adequate antimicrobial therapy, is estimated to be under 6 h, but should ideally be less than 1 h [6,7].

In this chapter, extreme POC testing will be referred to as POC diagnostic strategies that are or should be performed in low-resource settings, developing countries, or tropical areas where diagnostic infrastructures are often minimal or inexistent, and in situations following emergency crises or natural disasters where a minimal but efficient and clinically-relevant healthcare infrastructure must be rapidly put in place to avoid the onset (or minimize the expansion) of epidemics and limit the death toll [8,9,10,11,12]. From this point onwards, these areas will be designated “extreme environments”.

The current market for the POC testing of infectious diseases, in developing and developed countries, is largely dominated by immunology-based (lateral flow immunochromatographic; LFI) tests for the rapid detection of microbial antigens, tests that can be performed outside of laboratories but often displaying low sensitivity and specificity [12,13,14,15,16]. In many instances, LFI tests may provide diagnostic results sufficient to guide a healthcare professional towards timely and appropriate patient management measures but, in light of the prevalence of (increasingly resistant) transmissible diseases, such as tuberculosis (TB), where culture-based methods may require weeks-to-months for confirming the identification of the microbial culprit and providing information about its potential for multiple or extensive drug resistance, populations living in these regions or exposed to transmission would greatly benefit from the implementation of POC molecular tests enabling a more rapid detection of nucleic acid biomarkers of genetic targets [17].

### 1.1. The ASSURED Criteria and the Realities of Extreme POC Testing

The “ASSURED” (Affordable, Sensitive, Specific, User-friendly, Rapid, Equipment-free, Delivered to those in need) criteria have been proposed by the World Health Organization (WHO) to orient the development of diagnostic tests deployable in resource-limited settings [18,19]. It is estimated that tests complying with the ASSURED criteria and delivering an analytical performance of 85%–95% sensitivity and 85%–95% specificity could dramatically reduce to impact of infectious diseases in developing countries [15,19].

While simple rapid diagnostic tests already on the market may conform to the ASSURED criteria, the situation is quite different in the case of nucleic acid-, microfluidics-, and/or nanotechnology-based technologies where issues related to cost, user-friendliness, use of sophisticated instruments, and tighter environmental control of heat and humidity are seldom raised to slow down or oppose their implementation. Taking into account the fact that published studies generally focus on the analytical performance of POC tests in low-resource settings rather their effect on patient outcomes, Drain and colleagues [11] have suggested a revision of the ASSURED criteria of an ideal diagnostic POC test, in a fashion which may favor the implementation of tests with a higher technology content:

1. allows a quick clinical decision;
2. can be used at clinical point-of-care by health workers;
3. affordable;
4. rapid (yield a result during a clinic visit or within a reasonable period of time);
5. acceptable test efficacy;
6. cost effective.

Apparently circumventing the ASSURED criteria and in an effort to better diagnose and control infectious diseases such as human immunodeficiency virus/acquired immunodeficiency syndrome (HIV/AIDS), sexually-transmitted infections, and tuberculosis in developing countries, the WHO and the Foundation for Innovative New Diagnostics (FIND) have been supporting the benchmarking of rapid molecular diagnostic tests like the Xpert^®^ MTB/RIF of Cepheid (Sunnyvale, CA, USA) [1,2,3,12,20,21,22,23]. The downside of increased access to molecular diagnostics of TB is that the capacity of management and treatment of drug-resistant forms of the disease have not significantly augmented [24].

### 1.2. Challenges of (and Solutions to) Performing Analytical (Bio)Chemistry in Extreme Environments

In extreme environments, a number of elements and conditions may affect the performance and integrity of both the instrument and test components (disposables and reagents stored therein) and, hence, influence the precision and robustness of chemical or biochemical analysis of clinical samples [12,25]. These elements are applicable to a wide range of analytical methods and technologies for assessing chemical analytes (ions), antigens, and proteins (LFI tests and enzyme assays), as well as nucleic acids (molecular amplification). Realistically, healthcare authorities and providers of diagnostic technologies work together to overcome the most critical operational challenges of extreme POC testing. Thus, before investing on high-end equipment, it would be logical that reasonable investments be made to build laboratory infrastructures maintaining near-optimal conditions for most tests (temperature and humidity within diagnostic device and equipment tolerances), such that the risk of test or instrument failure is diminished. However, despite the best intentions to develop an adequate capacity for POC testing, human, cultural, and societal issues may provide additional barriers to adoption [1,26].

When it is envisaged to perform diagnostics at POC or in decentralized (satellite) laboratories, the ultimate goal should be an absolute simplification of diagnostic technologies so that they could be performed under any conditions or in any site. However, one needs to be practical and, depending on the disease(s) to diagnose at a particular site and on the technology(ies) most suitable within the limits of a diagnostic device/instrument and amplification/detection technologies, we should consider making modifications to surrounding infrastructures to adapt to the needs instead of trying to exceed the capacity of the technology (and risk diagnostic failure). We, thus, propose here a few principles to illustrate this difficulty. Moreover, we need to remember that using multiple technologies and different infrastructures will most likely be necessary as a simple approach will never fulfil all needs.

#### 1.2.1. Environmental Conditions Shall Dictate the Design of a Diagnostic Laboratory Infrastructure

In extreme environments, heat in excess of 40 °C, freezing temperatures, and humidity in excess of 70% are conditions which may severely compromise the efficiency of electronic components of instruments and affect the performance of protein-based reagents, even those containing heat-stable reagents or isothermal amplification tests. Alleviating the impact of these elements on the performance of diagnostic tests and of the laboratory staff will result in a more clinically- and cost-efficient infrastructure. This is not a trivial issue, but providing the laboratory with a good capacity for cold storage would be highly recommended to protect test components from harsh and/or highly variable conditions falling outside the operational limits determined by manufacturers, conditions which have been shown to affect the performance of rapid diagnostic tests (RDTs) [27], glucose meters, and blood analyzers [25,28]. To further protect test components from the effects of high humidity, disposable cartridges, cassettes, or devices should be provided individually in sealed packages [9]. Alternatively, POC tests could be performed in enclosures designed to control temperature and humidity [29].

The laboratory infrastructure, mobile or not, should provide a clean (dust- and insect-free) environment for performing POC tests, even those that can be performed within self-contained (sample-to-answer) devices. For example, the concept and operational requirements of a POC laboratory implemented in rural Senegal have been well documented by Sokhna and colleagues [30]. If possible, the infrastructure should have separate rooms for reagent preparation (clean room) and for thermal cycling and detection of amplification products (amplicons). Nonetheless and as suggested earlier, it would be strategically, economically, and medically beneficial if the diagnostic laboratory was designed upfront with ventilation and air conditioning support maintaining the infrastructure within the operational tolerances of tests and equipment, to relieve most sources of human and equipment stress [31].

In many developing countries and in regions afflicted by disasters, providing a stable electricity supply might be problematic and frequent power fluctuations or outage can damage the equipment or influence or stop the amplification cycles. For a more stable power supply, power conditioners or uninterruptible power sources (UPS) units should be used to maintain the operation of instruments and (self-contained) computers during electrical fluctuations/outages, until power is restored or until backup electricity is provided by batteries, solar or wind power, or by a fuel generator.

#### 1.2.2. Analytical Instrumentation

Analytical instruments should be selected on the basis of the configuration of the infrastructure and the anticipated throughput of tests, but smaller, more portable, and less energy-consuming instruments should be favored.

#### 1.2.3. Human Resources

The menu of tests offered by a particular infrastructure and their technical complexity will directly influence on the selection of laboratory staff. If “sample-to-answer” tests are used, the technical skill requirement would be lessened.

#### 1.2.4. Quality Control and Assurance

Each test should include a process control enabling to determine at which step (extraction, amplification, *etc.*) a test could have failed. Remote quality control should be engineered in the instrument as service visits may not be practical.

#### 1.2.5. Data Transmission

Electronic data transmission to computers or mobile phones should be implemented but the instrument should also provide a user-friendly interface for retrieving test results.

#### 1.2.6. Waste Disposal

Plastic waste should be minimized and proper arrangements should be made with local authorities for the management and disposal of potentially infectious biological materials. The use of biodegradable plastics could be envisaged if some of these new materials are amenable to (micro)fabrication and resistant to device assembly processes, but resorting to compact diagnostic technologies/devices capable of analyzing small volumes of clinical samples (<1 mL) would be more logical.

### 1.3. Nucleic Acid Amplification Technologies Amenable to (Extreme) POC Testing

A key event in the history of rapid diagnostic testing for infectious diseases was certainly the approval by the United States Food and Drug Administration of the IDI StrepB™ test, the first real-time PCR (rtPCR) assay approved for the rapid detection of *Streptococcus agalactiae* in parturient women [32]. This test was followed by several others marketed by Infectio Diagnostic (IDI) Inc. (now BD Diagnostics GeneOhm of Québec City, QC, Canada) [33]. These tests, along with five other assays developed by the *Centre de recherche en infectiologie de l'Université Laval* sold in over 50 countries, are part of the arsenal of PCR-based tests that have been approved by United States of America Food and Drug Administration (FDA) for the rapid nucleic acid-based diagnosis of infectious diseases since 2001.

Developed to circumvent the complexity, power and cold storage requirements, and cost associated to PCR thermal cycling (reagents and equipment), isothermal methods such as helicase-dependent amplification (HDA), loop-mediated amplification (LAMP), nucleic acid sequence-based amplification (NASBA), recombinase polymerase amplification (RPA), strand displacement amplification (SDA), and enzyme-assisted target recycling (EATR) have been exploited to develop nucleic acid detection assays combined with lateral flow-like or luminescence detection, several of which have been approved by the FDA for *in vitro* diagnostics [34,35,36,37,38]. Isothermal amplification methods may detect a broader range of microbial targets than rapid LFI tests, but the number of targets per test is generally less than the number achievable by multiplex PCR amplification.

The year 2015 will be remembered as a cornerstone year in the history of nucleic acid-based POC testing since four tests based on isothermal nucleic acid amplification and PCR-based amplification have been granted a CLIA-waived status. First, the POC detection of the influenza A and B viruses is now possible through the use of tests commercialized by Alere (Alere™ i Influenza A and B test, approved in January 2015, Waltham, MA, USA) and Roche (cobas^®^ Influenza A/B approved in September 2015, Basel, Switzerland). Second, those companies also received a CLIA-waiver for their rapid POC tests for the detection of *Streptococcus pyogenes* (group A streptococci), the cobas^®^ Strep A (approval in May 2015) and Alere™ i Strep A (approval in July 2015). While the Alere™ i tests rely on isothermal amplification using the Molecular. In Minutes™ (MIM) technology, the cobas^®^ tests are based on the Liat PCR-based amplification platform, initially developed by IQuum Inc. (Marlborough, MA, USA). In a sense, these accomplishments are quite important for extreme POC testing since they suggest that relatively complex molecular tests can be theoretically executed by skilled and less skilled healthcare staff.

### 1.4. Nucleic Acid-Based Extreme POC Testing: Simplex, Multiplex, or Multiparametric?

Bloodstream (sepsis), respiratory and urinary tract, gastrointestinal and healthcare-acquired infections are infectious syndromes (or syndromic infections) which impose an additional level of complexity to patient management due to their polymicrobial and/or multimicrobial nature [7]. In addition, these infections may be caused by resistant microorganisms such as carbapenemase-producing (also known as carbapenem-resistant) enterobacteria (CPE/CRE), *Acinetobacter baumanii* and *Pseudomonas aeruginosa*, or methicillin-resistant *Staphylococcus aureus* (MRSA) bearing antimicrobial resistance biomarkers that cannot be detected by immunology-based tests [39,40,41,42]. To guide the onset of an adequate antimicrobial therapy, POC tests enabling the multiplex or multiparametric detection of a panel consisting of the most relevant pathogens of a particular syndromic infection and associated antimicrobial resistance biomarkers would improve the chance of obtaining genomic information of clinical significance; needless to say, that rapidly determining if an infection is caused by a viral, bacterial, fungal, or parasitic pathogen would contribute to more efficient antimicrobial stewardship and better control of resistance gene evolution and dissemination. In a survey conducted to examine to better define diagnostic devices to resolve technological gaps for POC emergency and environmental disaster testing, respondents preferred a device capable of processing a single patient sample for multiple pathogens instead of testing many patients for a single pathogen, although there were few approved multiparametric tests available at that point in time [8]. For the time being, multiplex or multiparametric detection (in extreme environments) could be strategically done more efficiently with PCR-based platforms rather than isothermal amplification.

In developing countries however, the increased prevalence of multidrug and extensively drug-resistant tuberculosis has created a situation where implementing molecular diagnostics at POC, in fact at near POC, could curb the progression of the disease [17,22,23,43,44]. For diagnosing tuberculosis, it is well known that the smear microscopy method has a limited sensitivity and cannot distinguish between drug sensitive and drug-resistant *Mycobacterium tuberculosis* and other mycobacteria of the *M. tuberculosis* complex. Furthermore, the determination of antimicrobial resistance by culture-based methods requires weeks to months to produce results, thus contributing to increase the risk of household and community disease transmission.

## 2. Microfluidics, from Paper-Based Tests to the Innovative GenePOC Centripetal (Centrifugal) Platform

Current microfluidic platforms developed for biomedical applications can be broadly separated in two large groups: 1° capillary-driven devices such as lateral flow tests and paper-based devices and 2° fluidic devices where microfluid transport (pumping) is accomplished by more or less complex electroosmotic, electrochemical, pneumatic, electrohydrodynamic, acoustic, magnetohydrodynamic, or centripetal (centrifugal) mechanisms while microfluid control (valving) is performed by passive (check, capillary burst, or siphon) and/or active (pneumatic, phase-charge, or magnetic) valves [45,46,47].

### 2.1. Capillary-Driven and Paper-Based Microfluidics

Lateral flow immunochromatographic tests currently dominate the market of rapid diagnostic tests for infectious diseases [16]. Rapid LFI tests are relatively robust capillary-driven devices essentially fabricated using nitrocellulose and plastic materials. However, the demonstration that a paper matrix could be functionalized with hydrophobic polymers, to create functional channels and reaction chambers enabling the rapid and economical detection of analytes, has opened a new field of investigation and exciting possibilities for low-cost POC diagnostics [48,49,50]. It is anticipated that the maturation of paper-based diagnostics may provoke a revolution in extreme POC testing, but this revolution, similar to what has been achieved with integrated fluidic devices, will require the integration of the critical steps of nucleic acid-based molecular diagnostics, notably sample preparation, nucleic acid extraction and, most probably, isothermal amplification and target detection, onto low-cost paper devices [49].

### 2.2. Centripetal Microfluidics Principles and Recently Developed Integrated Devices

For more than 20 years, the field of fluidics has provided life science and biomedical research highly interesting applications through advances in microfabrication, microvalve design actuation, micropumping schemes, and implantable microelectronic components. Research and development efforts have yielded integrated platforms capable of performing fast and compact, ultimately “sample-to-answer”, devices minimizing human interventions, as well as contamination of the testing environment [51,52].

In a microfluidic centripetal (centrifugal) device (MCD; also designated as lab-on-a-CD due to the overall structure of most devices), fluids are displaced between chambers and/or reservoirs through microchannels (generally <100 μm in diameter or width) using the centrifugal force generated by rotating the device (reviewed in [45,46,53,54]). In a MCD, the transfer of microfluids is generally controlled by passive (hydrophilic, hydrophobic, or siphon) valves; a notable exception came in the form of ice valves implemented in the device described by Amasia and colleagues, to constrain a PCR reaction mixture during thermal cycling [55,56]. By design, it is possible to create various types of channels, chambers, and valves which can timely execute a bioanalytical process onboard the device, taking advantage of the fact that a MCD platform does not require an external pumping system. In a MCD system, the main actuator is a rotary motor used mainly to provide the acceleration needed for displacing fluids between compartments and open passive valves at relatively precise (burst) speeds; if necessary, bi-directionality of rotation can be programmed for mixing reagents or to exploit the pseudo-Coriolis force to direct a fluid volume to a particular compartment [57].

Interestingly, Sin and colleagues [46] have evaluated the properties of several microfluidic platforms, such as portability, throughput, instrument cost, capacity for multiplex analysis, diversity of microfluidic operations, accuracy, and programmability, and have confirmed the advantages and disadvantages of capillary-driven (lateral flow) platforms. However, their analysis suggests that, at an average cost, centripetal microfluidics have a better or similar capacity for multiplex analysis, diversity of microfluidic operations, and throughput of accurate tests than platforms with more complex pumping mechanisms. For extreme POC testing, MCD have the added advantage over capillary-driven devices that they are closed devices with minimal interactions with the environment.

### 2.3. Integrated Centripetal (Centrifugal) Microfluidic Devices

A recent survey of the literature has uncovered a highly-promising accumulation of integrated MCD developed for molecular diagnostics. We believe that these achievements illustrate the adaptability of centripetal microfluidics and the advances provided by significant improvements in microfabrication technologies. To suggest a potential for (extreme) POC diagnostics, it must be noted that many MCD platforms have been engineered to perform the following nucleic acid-based amplification methods:

1. PCR amplification [56,57];
2. rtPCR amplification [4,58,59];
3. reverse transcriptase real-time PCR (RT-rtPCR) amplification [60];
4. digital droplet PCR [61];
5. isothermal nucleic acid amplification [62,63,64,65,66,67].

Currently, the only commercialized centripetal microfluidic platform enabling an integrated rtPCR-based workflow for diagnostics of microbial infections is the Simplexa™ of Focus Diagnostics (Cypress, CA, USA). For extreme POC testing, we believe that the 8-well format would provide a more adequate (random access) configuration than the 96-well version of the platform.

Considering that the focus of this Special Issue of *Micromachines* is to demonstrate the potential impact of centripetal microfluidics on extreme POC testing, we would like to highlight several features of integrated devices which make these systems amenable to extreme POC testing:

1. many devices, developed to operate “sample-to-answer” assays, are being assembled with onboard dried reagents, a feature that is necessary to reduce the dependency to cold storage and increase shelf life of the device and its consumables. For some tests however where a conditioned sample cannot be used directly to rehydrate reagents, it will be necessary that other reagents, such as water or buffer used to elute analytes or polyenzymatic cocktails, be supplied in the liquid state. Resolving this challenge will require cold storage and technological solutions such as temporary containers, effective (leakproof) world-to-chip interfaces, or more robust microvalves for long-term storage in the device to avoid human interventions;
2. the implementation of isothermal amplification protocols should relieve some of the power constraints attributable to thermal cycling. However, thermal control (homogeneity) might still be required for ensuring the robustness of assays when performed in environments where conditions fall outside the tolerances suggested by the manufacturer [50];
3. the inherent multiplex capacity of rtPCR (and RT-rtPCR) amplification suggests that MCD platforms could be designed for the multiparametric assessment of the most prevalent pathogens belonging to microbial panels associated to respiratory, urinary, or gastrointestinal infections for examples. Multiparametric detection also increases the probability of detecting the microbial pathogen(s) and/or antimicrobial resistance biomarkers present in a biological sample collected from a patient displaying the symptoms of a syndromic infection [7]. Demonstrating the potential of rtPCR (or RT-rtPCR) implemented onto MCD platforms to control syndromic infections in extreme environments could provide useful information to guide the entrance of hybridization-based molecular diagnostic systems capable of simultaneously detecting more pathogens and/or resistance biomarkers per assay, but these post-PCR technologies are more cumbersome.

### 2.4. GenePOC, a Centripetal Microfluidics Platform Applied to Infectious Disease Diagnostics

The GenePOC Diagnostics platform is the culmination of a successful academic-industrial transdisciplinary research program in microfluidics, led by Prof. Michel G. Bergeron of the *Centre de recherche en infectiologie de l’Université Laval* (Québec City, QC, Canada), aiming to bring rapid nucleic acid-based testing at point-of-care [4,33]. GenePOC Inc. (Québec City, QC, Canada) was established in 2007 to develop and industrialize a microfluidic centripetal platform enabling a rtPCR or RT-rtPCR molecular diagnostic workflow for detecting microbial pathogens and/or antimicrobial resistance biomarkers from a wide range of clinical samples, many of which being potentially highly useful for extreme POC testing, namely nasal, rectal, vaginal, and throat swabs, as well as stool (liquid and soft) and urine.

#### 2.4.1. The GenePOC Disposable—An Integrated Sample-to-Answer Lab-on-a-Chip Device

**Figure 1:**
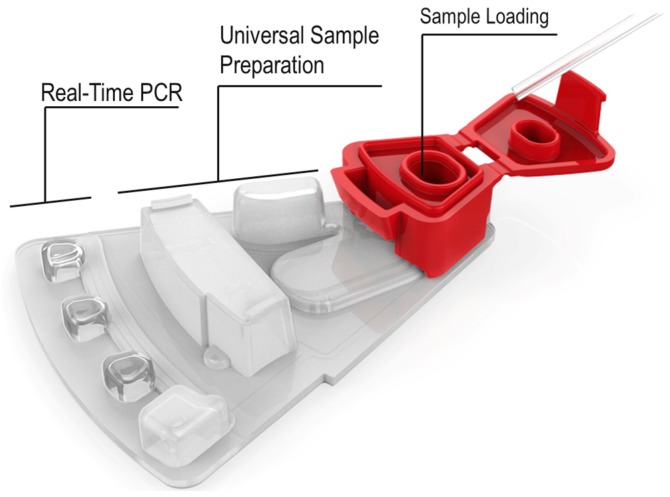
Schematic description of the GenePOC disposable, a MCD integrating the essential functions and components for performing nucleic acid-based diagnostics at POC. Copyright © GenePOC Inc. 2016 All rights reserved.

For the time being, the GenePOC Diagnostics platform distinguishes itself from most of the devices listed in Section 2.3 by offering a highly compact disposable device (and instrument), produced with onboard stabilized (dried or liquid) reagents, and displaying a very good potential for multiparametric analysis (up to twelve [12] targets per test) (see Figure 1). We believe that these features make the platform as applicable to extreme POC testing as the GeneXpert instrument, but to a lesser cost. Because of all the microfluidic engineering programmed onboard the device, in the form of the network of chambers and channels controlled essentially by centripetal actuation and simple fluid metering features, the GenePOC disposable is the real core of the platform.

The GenePOC disposable (Figure 1) is composed of three operational sectors:

1. **A world-to-chip interface** used for the manual loading of 100 to 200 μL of clinical sample conditioned in GenePOC sample buffer; upon loading, the chamber is sealed by a lid to minimize the release of potentially contaminated liquids/aerosols in the instrument or laboratory environment. Conditioned clinical samples that have been successfully tested in the plastic disposable include nasal, rectal, vaginal, and throat swabs, as well as stool (liquid and soft) and urine; these samples could be used to diagnose a wide array of (syndromic) infections which are prevalent in low-resource settings or known to threaten populations in areas under emergency crises or confronted by natural disasters. Blood testing, a Holy Grail of clinical microbiology, cannot yet be accomplished on the platform since this matrix would need off-chip sample preparation including microbial pre-concentration. Once the conditioned sample is loaded in the disposable with a transfer pipette, the disposable is then ready to be placed into the rotor of the instrument which is designed to simultaneously handle (and automatically balance) 1 to 8 disposables during runs performed at rotation speeds up to 3500 RPM. The GenePOC instrument acts as a centrifuge to displace fluids between chambers and compartments of the disposable device, through a series of siphon and passive valves which can be primed or actuated by centripetal acceleration. Upon starting a run, the initial programmed centrifugation will direct the conditioned sample to the sample preparation chamber which is designed to direct excess sample to a waste reservoir before the next step;
2. **The universal sample preparation sector** enables two (2) critical bioanalytical steps, nucleic acid extraction followed by distribution of a metered volume of nucleic acid extract into a dilution chamber. After a quick centrifugation where excess conditioned sample is directed to a waste chamber, the remainder volume of conditioned clinical sample is homogenized and nucleic acids of the microbial cells of the sample and of a microbial process control (already in the chamber) are mechanically extracted by the shearing forces generated by the magnetically-actuated horizontal movement of a metallic disk through a slurry of glass beads. After a short centrifugation, a metered volume of nucleic acid extract is directed to the dilution chamber where PCR inhibitors are inactivated by heating at 95 °C and the ionic conditions of the nucleic acid extract are adjusted to optimize rtPCR (or RT-rtPCR) amplification;
3. **The molecular amplification and detection sector** is the only portion of the disposable device where assay-specific reagents (thermostable DNA polymerase(s), primers, and fluorescent detection probes) enabling target amplification by rtPCR or RT-rtPCR are stored in the dried state. These reagents, aliquoted in three independent PCR chambers, are optimized to perform rtPCR assays generally displaying an analytical limit of detection of 5–10 copies per reaction. After inactivation of PCR inhibitors and adjustment of ionic conditions in the dilution chamber, the nucleic acid extract is distributed in the chambers of the sector where rehydration of reagents and nucleic acid amplification will occur. As the detection system of the GenePOC instrument is designed for the concomitant excitation and light collection of four fluorophores with non-overlapping spectra while the device is rotating, an assay implemented in a GenePOC disposable device can be designed to detect up to 12 genetic targets, including that of the microbial process control, when the three PCR chambers are used to their fullest.

#### 2.4.2. The GenePOC Workflow and Its Potential for Extreme POC Testing

**Figure 2:**
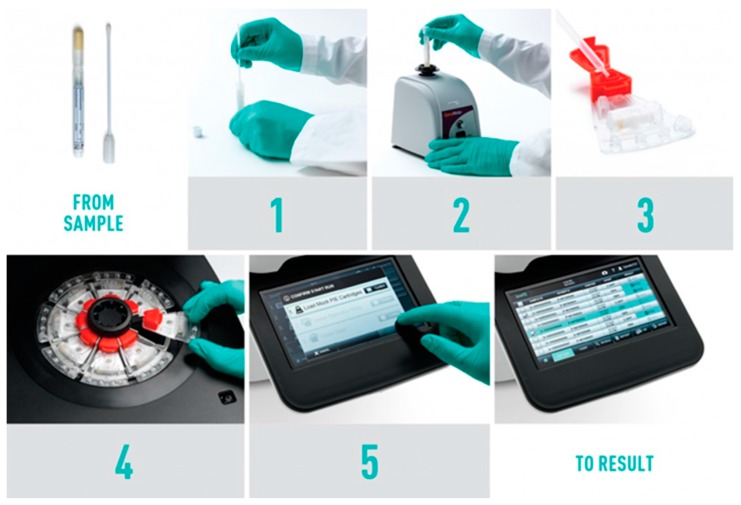
The workflow of the GenePOC platform. Source: GenePOC Inc.

The GenePOC Diagnostics instrument is essentially an air thermal cycling mini-centrifuge designed to simultaneously perform up to eight (8) independent rtPCR-based tests (see Figure 2). The workflow can be dissected as follows:

1. the clinical sample, a swab, a loop of stool, or alternatively a volume of liquid sample, is mixed with a proprietary sample buffer. For operation at POC, we believe that the collection and handling of those simple samples can be done, even in low-resource settings, by skilled and less skilled healthcare workers;
2. the clinical sample is conditioned (homogenized) by vigorous mixing;
3. a transfer pipette is used to load 100-200 μL of conditioned sample in the specified GenePOC disposable. The flexible volume range that a disposable device can accept imposes less technical stress to healthcare workers since the design of the microfluidic device precisely controls the amount of sample to be treated and from which the nucleic acids are extracted. The disposable device is also designed to deliver a precise amount of nucleic acid extract to the dilution chamber;
4. after the original sample container and the corresponding loaded disposable are scanned by the instrument barcode scanner, the disposable is placed in the instrument holder. As GenePOC pathogen/panel-specific assays are developed with generic thermal cycling protocols, the instrument can run up to eight (8) different tests simultaneously, thereby providing a very good flexibility with regards to diagnostic test selection and number of tests per run;
5. when the lid is closed, the instrument starts the test(s) run;
6. generally, in less than one hour, the results are displayed by the instrument. Results can be printed out or transmitted via various connectivity interfaces.

In light of the recommendations that laboratories operated in extreme environments should be capable to maintain temperature conditions within the tolerances of electronic instruments, and that PCR-based tests can provide a capacity for multi-parametric detection, we believe that a platform such as GenePOC could become a clinical tool of high value for extreme POC testing and find its rightful place in highly delocalized clinical microbiology testing. Contrary to most platforms listed in Section 2.3., the GenePOC POCT solution is already more compact in size (less plastic waste than other testing solutions), more mature, technologically, and more adaptable to the testing of a wide array of microbial pathogens and antimicrobial resistance biomarkers, not only from many different types of clinical samples but also by the fact that convergent thermal cycling conditions render the simultaneous testing for different pathogens and resistance biomarkers possible, in independent disposable devices.

## 3. Conclusions

The environmental conditions prevailing in low-resource settings, developing countries, tropical areas, or during emergency crises or natural disasters exert significant stress on populations, emergency relief, and healthcare workers, as well on the reagents, tests, and instruments used to diagnose infectious diseases, in order to contain or control epidemics. In this work, we have browsed a portrait of these conditions and stresses and illustrated the potential of centripetal microfluidics for highly efficient nucleic acid-based diagnostics of infectious diseases through rapid identification of microbial pathogens and of the antimicrobial resistance genes they may carry.

One such centripetal microfluidic platform, developed by GenePOC Inc. of Québec City, is primarily designed to provide a workflow compatible with conditions prevailing at (near) POC and in delocalized settings. In large healthcare institutions (hospitals) where diagnostic activities are becoming more centralized, we envision that GenePOC instruments and other POC tests of high clinical utility could be operated in near POC satellite laboratories by skilled or less skilled healthcare workers, at the emergency or in specific departments (obstetrics or intensive care units) frequently requiring *stat* microbiology. In smaller institutions, laboratories of outpatient clinics or pharmacies, or satellite or emergency laboratories operating in extreme environments, the GenePOC tests displays functional and operational characteristics which would make it suitable and adaptable for use in extreme environments, notably a universal sample preparation process efficient with a wide range of biological samples, and onboard dried reagents for rtPCR amplification of up to 12 genetic targets, all of this on a self-contained disposable plastic device. The impact of a technology like GenePOC on extreme POC testing could undoubtedly be realized if the satellite or emergency laboratories provide a reasonable control of temperature and humidity. Thus, moving from the comfort of our clinical laboratories to those of remote installations devoted to control infectious diseases in extreme environments would be a great accomplishment for the health of humankind.

## Abbreviations

The following abbreviations are used in this manuscript:

CLIA	Clinical Laboratory Improvement Amendments of 1988
CPE	carbapenemase-producing enterobacteria
CRE	carbapenem-resistant enterobacteria
EATR	enzyme-assisted target recycling
FDA	Food and Drug Administration (United States of America)
FIND	Foundation for Innovative New Diagnostics
HDA	helicase-dependent amplification
HIV/AIDS	human immunodeficiency virus/acquired immunodeficiency syndrome
LAMP	loop-mediated amplification
LFI	lateral flow immunochromatographic (tests)
MCD	microfluidic centripetal (centrifugal) device
MIM	Molecular. In Minutes™
MRSA	methicillin-resistant *Staphylococcus aureus*
NASBA	nucleic acid sequence-based amplification
PCR	polymerase chain reaction
POC	point of care or point-of-care
RDT	rapid diagnostic test
RPA	recombinase polymerase amplification
RT-rtPCR	reverse transcriptase-real-time PCR
rtPCR	real-time PCR
SDA	strand displacement amplification
TB	tuberculosis
UPS	uninterruptible power source
WHO	World Health Organization
